# Challenges in Translational Research: The Views of Addiction Scientists

**DOI:** 10.1371/journal.pone.0093482

**Published:** 2014-04-04

**Authors:** Jenny E. Ostergren, Rachel R. Hammer, Molly J. Dingel, Barbara A. Koenig, Jennifer B. McCormick

**Affiliations:** 1 Department of Health Behavior and Health Education, University of Michigan, Ann Arbor, Michigan, United States of America; 2 Biomedical Ethics Research Unit and Mayo Clinic College of Medicine, Rochester, Minnesota, United States of America; 3 Center for Learning Innovation, University of Minnesota Rochester, Rochester, Minnesota, United States of America; 4 Departments of Social & Behavioral Sciences, and Anthropology, History, and Social Medicine, University of California San Francisco, San Francisco, California, United States of America; 5 Divisions of General Internal Medicine & Health Care Policy Research, and Biomedical Ethics Program, Mayo Clinic, Rochester, Minnesota, United States of America; Penn State College of Medicine, United States of America

## Abstract

**Objectives:**

To explore scientists' perspectives on the challenges and pressures of translating research findings into clinical practice and public health policy.

**Methods:**

We conducted semi-structured interviews with a purposive sample of 20 leading scientists engaged in genetic research on addiction. We asked participants for their views on how their own research translates, how genetic research addresses addiction as a public health problem and how it may affect the public's view of addiction.

**Results:**

Most scientists described a direct translational route for their research, positing that their research will have significant societal benefits, leading to advances in treatment and novel prevention strategies. However, scientists also pointed to the inherent pressures they feel to quickly translate their research findings into actual clinical or public health use. They stressed the importance of allowing the scientific process to play out, voicing ambivalence about the recent push to speed translation.

**Conclusions:**

High expectations have been raised that biomedical science will lead to new prevention and treatment modalities, exerting pressure on scientists. Our data suggest that scientists feel caught in the push for immediate applications. This overemphasis on rapid translation can lead to technologies and applications being rushed into use without critical evaluation of ethical, policy, and social implications, and without balancing their value compared to public health policies and interventions currently in place.

## Introduction

The belief that the goals of understanding and use are inherently in conflict, and that the categories of basic and applied research are necessarily separate, is itself in tension with the actual experience of science. –Donald Stokes in Pasteur's Quadrant [1] ^(p12)^

Between fiscal years 1998 and 2003, the budget of the National Institutes of Health (NIH) doubled with hopes and promises of medical breakthroughs of cures and treatments. On the tail of this, in 2003 the National Human Genome Research Institute announced the completion of the Human Genome Project. [2] This ambitious undertaking was a fundamental achievement for basic scientific understanding of human biology. It also ignited a “biological revolution,” launching the life science research community into the “genomic era” with its promise of technological advances for the practices of medicine and public health. [3]

Much emphasis has accordingly been placed on the “translation” of research findings from “bench to bedside and beyond.” In 2003 the NIH unveiled its Roadmap initiative, calling for a highly-mechanized and multi-disciplinary translational approach to accelerate the pace of discovery and expedite the movement of new knowledge into public benefits.[4] Two years later, the NIH created the Clinical and Translational Science Awards (CTSAs), aimed at improving health by encouraging innovative ideas, research collaboration, engagement with the non-scientific community, and accelerated processes of translation.[5] More recently, in a 2012 TEDMED talk, NIH director Francis Collins emphasized the need to speed the process of translation by bridging the “yawning gap” between basic knowledge about the causes of disease and the development of novel treatments.[6] He likened the “reality” of the translational process to a sailboat, tugboat, and swimmer crossing a river so hazardous that only with luck would one make it from the bank of “fundamental knowledge” to the other side—“application.”

To close the gap between scientific discoveries and health impact, and to prevent basic research findings from becoming “lost in translation,”[7], [8] a variety of translational models have been proposed. Translation has been described as a “bench to bedside” model,[9] a two-phase process,[10] a continuum with five T-phases (T_0_-T_4_),[11] and a process of three translational (3T's) phases.[12] It could perhaps be argued that translational research is merely a re-packaging of the term “applied research,” which has been in the lexicon for over fifty years.[1] The term has also been used to refer to the relevance of basic science to public health practice; the emerging field of public health genomics has focused on facilitating the translation of genomic discoveries into population-level benefits by applying genetic information to disease prevention efforts.[13], [14] However, efforts to translate genetic and other research findings into public health and clinical practice have met significant challenges,[15], [16], [17] rooted in the biological, social, and political complexity of human disease.

Addiction, which is a major public health problem with wide-ranging and devastating consequences for affected individuals, their families, society, and health care systems, fully illustrates this complexity.[18] Described as a “bio-cultural phenomenon,” addiction spans the boundaries between the fields of neuroscience and the psycho-social sciences.[19] Many genes, neurobiological pathways, and socio-environmental factors are implicated in substance-use initiation and dependence.[20] There is no simplistic, linear, cause-and-effect way to describe the etiology of addiction. A multi-disciplinary scientific approach is imperative to understanding the complex social, political, behavioral and genetic interactions that influence its development and is essential to the successful integration of basic research findings, including those from genetic research, into public health and clinical practice. Thus, addiction presents a good case example to examine in the context of translational science. Given the push for translation and its influence on the allocation of funding for basic research, it is also critical for scientists—basic, applied, and clinical—to think about the ways in which their discoveries might affect public health and society, as well as the ethical and policy issues involved in translation.

We report here the perspectives of addiction scientists on translation and how their work fits into the translational paradigm. The data are from a large study exploring the geneticization - viewing diseases, conditions, and behaviors as being determined all or in part by genetic factors - of addiction.[21], [22], [23], [24] The broader study captures the views of addiction patients as well as scientists' thoughts on the definition of addiction phenotypes and etiology. We examined scientist perspectives on the topic of translation because they are important stakeholders who possess a deep understanding of the science and its potential applications. There is also a need for the scientific community to be more engaged in discussions with the public and policy-makers concerning when and if discoveries should be translated. Ironically, given all the discourse about translational science, scant attention has been paid to the voices of those at the start of the pipeline, those whose work is being “translated.” Our study brings their voices into debates about translational science.

## Methods

### Ethics Statement

This study was determined exempt by the Mayo Clinic Institutional Review Board and the University of Minnesota Institutional Review Board. Verbal informed consent was obtained from all participants and documented in our research records.

### Participant Sampling

Our final sample consists of 20 scientists engaged in addiction research (Table 1). Using our prior research experience conducting semi-structured interviews, we tentatively set a goal of interviewing 20 scientists. No new themes were introduced and saturation was achieved by the 20^th^ interview, and we elected to not add additional scientists to our sample. Themes were repeated after the eighth interview.

**Table 1:** Description of Sample (N = 20).

				*N*
**Primary Research Area**				
	Genetics/Behavioral Genetics			7
	Neuroscience/Neurobiology			6
	Molecular Biology/Pharmacology			5
	Epidemiology			1
	Clinical Trials			1
**Model System**				
	Animal			11
	Human			9
**Institution**				
	Public University			9
	Private Non-profit Medical Center			5
	Private University			4
	Private Non-profit Research Institute			2
**Academic Rank**				
	Professor			16
	Associate Professor			3
	Assistant Professor			1
**R01 Grant Recipients**				17
**NIDA Funding**				12
**NIAAA Funding**				7

A list of potential participants was generated based on suggestions from the scientists on our project's multi-disciplinary advisory board and the attendees of recent NIDA Genetic Consortium meetings (http://www.drugabuse.gov/about-nida/organization/workgroups-interest-groups-consortia/genetics-workgroup-gwg/nida-genetics-consortium-ngc). [As NIDA funded investigators, twoof us (JBMc and BAK) regularly attended these meetings.] Our sample was selected to obtain a diverse range of backgrounds from the field of addiction research and a balance between those working with human and animal subjects.

### Procedures

Selected scientists were invited by email to participate in a 30–60 minute semi-structured interview covering a breadth of topics related to genetic research on addiction. All interviews were audio-recorded and transcribed, and participants were assured that no personal identifiers would be reported in any data presentation. This paper focuses specifically on responses to three questions: 1) What is the primary purpose of your research and how do you envision it fitting into the translational spectrum? 2) How much utility does this body of science have for addressing addiction as a public health problem? and 3) How do you think a link between genes and addiction might affect public attitudes toward drug use and addiction? The interview guide is available on request.

### Analyses

We used NVivo 8 software to catalogue and organize the interview data for synthesis and analysis. The research team analyzed data using standard qualitative processes of identifying common meanings and core consistencies and meanings or recurring regularities.[25] Though categorizations were partially driven by themes delimited in the interview guide, we also utilized strategies that allowed novel themes and meanings to emerge from the data.[26] After team members had independently read through all the transcripts and identified prominent themes that could be followed within any given transcript as well as across transcripts, the team met as a group to create specific labels that could be used to systematically code each transcript. The team agreed upon definitions of each label or code through an iterative process with the final product being a code book, available upon request. In some cases, a particular theme may actually be described by several codes. For example, some of the data from the two codes “Translational Pipeline” and “Utility of Biological Data for Public Health” form one of the themes presented while some of the data from the three codes “Translational Pipeline”, “Animal Model and Human Model—Advantages and Disadvantages”, and “General Funding for Addiction Research” all contribute to a second theme. Two team members then used this code book to independently code all transcripts. Discordances were resolved through a consensus process that included a third member of the research team and thus kappa analysis was not employed. We then used written memos to develop the concepts and categories from rudimentary representations of the raw data to more complex, dense, clear and accurate summations of analytic concepts. Memos are short narratives describing the data under a particular set of codes that the research team identified as coming together to describe the original theme identified. [26], [27] This approach allowed us to summarize the full range of responses, and to highlight particularly illuminating or representative quotations.

## Results

Interviews lasted on average 40 minutes in length with a range of 25 to 75 minutes. Three themes emerged from the data: 1) Of course our research translates!, in which participants expressed optimism about translation of their research; 2) Don't forget the value of basic science, in which participants pointed to the value of basic research findings; and 3) Problems with pushing translation too quickly, in which participants expressed their views about the major emphasis on translation. Overall, our participants believed that genetic research held promise in the clinic, but they also openly expressed reservations and concerns.

### Of course our research translates!

Over half of participants endorsed the view that their work could be translated into clinical or public health practice. Some made strong statements about this translational potential, although frequent use of futuristic language implied a rather long translational pipeline.

The most commonly identified translational route was from basic science research to pharmaceutical intervention. One participant saw his/her research as “very much translationally oriented…at the end of the day I would like to find something that leads to a drug” (Neurologist). While pointing out the limitations of genetic research and pharmaceutical interventions, another participant sought the development of a “pill” that would “make interventions at the psychological and social level more effective.” (Neuroscientist). Another imagined an eventual cure through “rehabilitating circuits” in the body that are not working, emphasizing that this would be a “major step forward to returning the person to society who will no longer be a public health burden” (Neurobiologist).

Several envisioned better prevention through population screening or targeted screening of patients seeking preventive care. One highlighted the potential of neurological markers, presenting a futuristic scenario in which individuals could go in for their annual physical to find out there is “too much CRF in [their] amygdala…” which would indicate that they were “…probably under a great deal of stress and may be vulnerable to alcoholism.” (Neurobiologist). Another scientist outlined a population-based strategy for preventing addiction including the capacity of “identifying populations at risk and targeting prevention to those individuals… [a] sort of joint assessment of genotype and environment.” (Behavioral Geneticist). Another hoped that genetic research would lead to new ways of determining individual risk for addiction:

- In a futuristic world when every human's genome sequence will be known and stored in their iPhone, we will be able to say, ‘okay, you have elevated risk for cocaine addiction by 2.3 fold based on these 30 genes variations in these 30 genes that you possess'… It would be nice if [this knowledge] is used to work with children when they are younger and identify ways in which the vulnerability to get into drug use can be redirected to more healthful activities. (Neuroscientist).

A few scientists talked about how biological research into the etiology of addiction could be used to educate the public. For example, one noted the importance of basic science knowledge discovered through addiction research for educating the public about the consequences of drug use. This individual said that “from a public health policy vantage,” this information can help to protect young adolescents from exposure to drugs because they will learn about the changes the brain undergoes as a result of using drugs. (Psychobiologist). A scientist pointed out how our understanding of fundamental kinetics actually translates into useful public health education about the dangers of binge drinking:

- What do you do when you gulp your alcohol? Well, you increase your blood ethanol concentration. [When this concentration] is really high. …it changes the whole kinetics of how much alcohol gets to your brain… that is a public health message I think. (Behavioral Neuroscientist).

Almost all interviewees saw biological research on addiction as having broad societal benefits, such as reducing stigma and self-blame and helping addicts to get over the initial stages of denial. One, for example, stated:

- I think it has done wonders in terms of trying to put addiction into the same realm of medicine as other diseases, like diabetes, which are bio-behavioral, or heart conditions. (Psychobiologist).

Many believed their research “might remove some of the kind of moralistic view of [addiction]” (Behavioral Geneticist) and were hopeful that it might help to de-criminalize and place addiction more properly in the realm of disease, rather than social transgression.

### Don't forget the value of basic science

Many were also eager to discuss the relevance of basic research to the larger issues in translational research policy. Participants hoped their research would be translated, but acknowledged that translation takes time, that bodies of knowledge are built slowly over many years, and that basic science has value even in the absence of swift translation. Some thought a drug rather than new knowledge is too often assumed to be the desired product of translational science. A scientist noted:

- You have to do the science [to] find a drug and that can come from chance, that can come from playing around with different drugs; different animal systems; that can come from genes. …We know a lot of the genes now, and will we ever have anything better than nicotine replacement [besides] increasing the tax of cigarettes? I don't know – maybe, maybe not. But I believe that you have to do the science because it is extremely interesting and it might lead to a new achievement, but it is not a given fact that it will. (Molecular Biologist).

This scientist went on to express caution about overselling the rapid translation of genomic research findings to the public, warning that, “you have to do the science and let it unfold” and, “you hope it will lead to something that is clinically relevant, but it really may not… you can't oversell to the public, especially; then your credibility goes down.” Another reasoned that while scientific applications can emerge from basic science by chance, translation is never guaranteed:

- If we are really fortunate, we would identify specific biological mechanisms, genetic mechanisms that you could [use to] develop a therapy… but now, because finding these specific mechanisms has been really hard…, it is much harder to say how that work will translate into benefiting for sure the life of any particular person who has an addiction problem. I still think it is important because … over the course of time, as you aggregate information across these research studies, you have a better idea of what to do next and how to get closer to sort of thistranslational endpoint. (Human Behavioral Geneticist).

One participant commented on the incongruity between his/her actual research objective and how he/she sometimes finds himself/herself framing his/her research. Though this person believed “curing all of these diseases” is a long-term goal, the individual also cautioned: “the reason for doing what we do is to understand how the brain works. And I think that will lead into insights that do allow for treatment, but I think most of the time it doesn't.” (Molecular Neurobiologist). The informant went on to argue that the information we accumulate from genetic research is interesting and important in and of itself, regardless of whether it leads to new treatments:

The way we are sequencing all these genomes right now for addiction, we are learning as collateral information which genes can tolerate mutations without causing apparent problems. And that tells us something about the evolution of our species… I don't know that those are really, really exciting topics to the average person that just wants a pill to treat their problems, but…I wouldn't say they are useless, in fact, I think they are pretty interesting and… are pretty important. But, it is kind of like the argument about telescopes. I mean, do we really need to know what the Hubbell Telescope shows? (Molecular Neurobiologist).

Though these scientists believe in the fundamental value of basic science, their responses suggest that the emphasis on rapid translation has created an uncomfortable tension for basic researchers, the same tension as noted by Stokes above.[1]

### Problems with pushing translation too quickly

Many of our participants had reservations about the increasing emphasis on translational science. One stated that he/she was “all for it, we need to translate…,” but also warned “…we can do harm by putting excessive focus on translation,” because the value of basic research may be overlooked leading to unfortunate consequences (Neurosceintist). Another warned that in the future NIH may push even harder for translation:

- I think another major thrust of NIH, in general, is going to be translational which is a big word that says not a lot of anything. But it is going to be translational with a more specific aspect to it, which is how can we convert all of this knowledge that we have… into better treatments for addiction. And I think that is coming. (Neurobiologist).

One participant expressed concern that the emphasis on translation may tempt researchers to misrepresent data in order to obtain funding:

- It would be nice if… everyone was quite clear about what they were doing and why and how the bridge from one could go to the other rather than people misrepresenting the data as being genuinely potentially translational just because that is what you have to say to get money. And I think that has become a bit of a problem. (Behavioral Neuroscientist).

A scientist thought the current translational paradigm should place more emphasis on how research questions are asked: “I think this bench to bedside model is wrong. It has to be integrated. You should be designing your experiments with the translation built in” (Molecular Geneticist). Another suggested that the current focus on translation comes from the public perception that NIH-supported basic research has not led to significant improvements:

- I think there has been such a big focus now on translation because, in general, we haven't done such a great job of making that translation…because of the perceived insufficient progress, there has been an increased attention to translation. (Neuroscientist)

Some interviewees felt that the public expects too much of the scientific community, and that technologies may be reaching clinical application too soon: “I'm a little bit worried that there is too much hope and too much emphasis placed on the application of these technologies.” (Human Behavioral Geneticist). None of our participants questioned the value of their work to the overall public health goals of improving addiction prevention and treatment over the long term; however, many questioned the usefulness of the push for quick translation and even the term itself.

## Discussion

The scientists we interviewed believe their research will lead to clinical and public health applications. They were able to posit many future practical applications of their work, such as new treatments, prevention strategies, and increased public understanding of addiction. However, they also believed that to bear fruit, the scientific process must be free to play out, that fundamental biological discovery should not necessarily be tethered to the promise of societal benefit. Our data suggest that many of our scientist participants feel caught in the push for immediate applications and have reservations about the value of emphasizing translation too soon.

High expectations that genetic science will lead to new prevention and treatment modalities for a wide range of diseases, including addiction [28], have exerted pressure on scientists and funding agencies to demonstrate the real-world benefits of large monetary investments in genomic science. The requirement that scientists describe the translational objectives of their research to get funding may have unforeseen consequences for the scientific process and, in turn, public welfare, as noted by some of our interviewees. The overemphasis on rapid translation can produce a situation where technologies and applications are being rushed into use without critical evaluation of ethical, policy, and social implications, and without regard to proven and successful public health policies and interventions currently in place, such as smoking bans or taxation.

Our interview data suggest that pharmaceuticals are often assumed to be the target outcome of translational science, and in the case of addiction, this privileging of drug therapies may have the detrimental effect of weakening social and behavioral interventions [29], [30] This may in turn limit social and behavioral research and impaire the serious exchange of ideas and information from different fields. This critical ethical issue exists not only in addiction research but also in research on other complex health conditions, such as diabetes and cardiovascular disease [31].

One interpretation of our data is that rather than placing so much emphasis on translating basic science from “bench to bedside and beyond,” funding agencies and policy-makers ought, as CTSA and similar policy moves have intended, to consider how to encourage more multi-disciplinary research. Social and behavioral science research that incorporates and examines elements of ELSI (Ethical, Legal and Social Implications of genetics) is extremely valuable to the successful evaluation and movement of genetic discoveries into social and health benefits. Indeed, some of this multi-disciplinary research is already happening through the CTSA Consortium as public health, clinical, and basic researchers collaborate to bring findings from bench to bedside. One example is the NIH-funded eMERGE Network, a collaborative and multi-disciplinary consortium that gives a key role to ELSI issues in conducting research that links genomic data with information from the electronic medical record.[32] Using similar strategies, the Clinical Sequencing Exploratory Research Program brings basic scientists and ELSI researchers together to explore the complexities raised by translating whole exome or genome sequencing into clinical application.[33] However, collaborative bridges between the natural/life sciences and social/behavioral sciences are not yet the norm, and are limited by traditional institutional structures, such as departments that maintain rigid silos and/or rely on investigator-initiated R01 grants as benchmarks for promotion. [34]

Our study has several limitations. Two members of the research team (JEO and JBMc) conducted the interviews, which may have limited the uniformity in how questions were posed. However, JEO observed a number of the interviews conducted by JBMc prior to conducting interviews, and following many of the interviews, JBMc and JEO met to debrief and review the interviewee's responses to questions. In addition, our findings reported here span all 20 interviews. The original objective of our interview study was to assess how scientists talk about and conceptualize genetics in the context of addiction, not to examine their perceptions of translational science. Operating within the framework of this main objective may have limited opportunities for further follow-up on some discussion points that were raised.

Nevertheless, we believe the fact that comments about translational science arose even in the context of genetics and addiction supports our conclusions. These data provide a starting point to further this investigation and may serve as a guide in designing additional research instruments; and future studies may benefit from exploring these qualitative themes in a larger sample using quantitative methods, for example a national survey. Finally, our data and conclusions are limited by the small sample size and our approach to creating our sample, and are not generalizable because of our use of semi-structured interviews. In contrast to quantitative approaches, which use data to test existing models, hypotheses, or theories, qualitative techniques are used to develop detailed understandings of phenomena from patterns found directly in the data. Like much qualitative research, our findings may lack the generalizability that larger and more random samples provide, but what is lost in terms of sample size and generalizability is gained in the depth and content validity of the findings, which put forth the views of scientists in their own words.

## Conclusion

Translation is often a long and arduous process, as noted by some of the interviewees. Impatience for immediate results may do more harm than good, forcing attention on quick fixes rather than comprehensive solutions. Neither policy-makers, funders, scientists nor the public are inclined to accept the fact that some fundamental discoveries will not easily lead to direct and immediate outcomes, such as patents or magic pills. With more collaborations between researchers making discoveries, “at the bench,” and those, “out in the field,” unforeseen solutions to bridge the gaps spanning basic research, public health, and social policy-making may be found.

Our current paradigm of studying the biology of disease in one silo, the environmental influences in another, and the psycho-social aspects as an unwieldy add-on, is not sufficient. We must acknowledge that the conditions we wish to remedy are as complicated as humans themselves. Although translational science has been offered as a potential solution to the problem of non-integrated research, disciplinary rigidity, and failures of knowledge transfer [7], [11], the data from these interviews suggest that scientists, policy-makers, and the public may have become lost in their own impatience with scientific progress and overly focused on finding “quick fix” treatments for complex disorders like addiction.
